# Editorial: “Inside-Out” vs “Outside-In” Paradigms in Multiple Sclerosis Etiopathogenesis

**DOI:** 10.3389/fncel.2021.666529

**Published:** 2021-03-01

**Authors:** Antonio Luchicchi, Paolo Preziosa, Bert 't Hart

**Affiliations:** ^1^Division Clinical Neurosciences, Department of Anatomy and Neurosciences, Amsterdam Universitair Medische Centra, Vrije Universiteit Medical Center, Amsterdam, Netherlands; ^2^Neuroimaging Research Unit, Division of Neuroscience, Istituto di Ricovero e Cura a Carattere Scientifico San Raffaele Scientific Institute, Milan, Italy; ^3^Neurology Unit, Istituto di Ricovero e Cura a Carattere Scientifico San Raffaele Scientific Institute, Milan, Italy

**Keywords:** multiple sclerosis, pathogenesis, outside-in, animal models, biomarkers, molecular aspects, inside-out

Multiple sclerosis (MS) is a chronic inflammatory/demyelinating disease of the central nervous system (CNS) (Filippi et al., 2018) of unknown etiology.

Two complementary paradigms, indicated as “*outside-in”* and the “*inside-out,”* are leading discussions about the MS origin (Stys et al., 2012). The outside-in paradigm posits a peripherally-elicited autoimmune attack against myelin as root cause for MS, whereas inside-out implicates a primary CNS cytodegenerative process alarming secondary autoimmune reactions against myelin debris.

Which of the two theories better depicts the very first pathological changes in MS is debated. Since a better evaluation of MS etiopathogenesis could also optimize patients' management, the present issue was aimed to stimulate a discussion about possible overlooked candidates in support of each theory. We collected nine contributions where authors discuss the *outside-in*/*inside-out* paradigms from different angles.

Three main aspects were discussed: (i) animal models of MS etiopathology, (ii) cellular processes in MS pathophysiology, and (iii) tools and biomarkers enabling investigation of *in vivo* pathophysiological mechanisms already from the prodromic/early phases of MS.

Titus et al. and Sen et al. summarized the most updated models supporting either the *outside-in* or the *inside-out* theory of MS. In particular, the first study concluded that, due to the heterogeneous manifestation of MS pathology, a combination of both paradigms, rather than one of the two, may better explain the origin of MS. Conversely, the second article supported the value of more recently developed models, like the cuprizone (CPZ) mouse, to investigate primitive changes occurring in MS brains. Interestingly, CPZ models seem ideal to reveal the mechanisms involved in MS origin before the immune attack intervenes, and to study MS progression in conditions of immune reaction and protection.

It is well-known that several genetic factors contribute to the increased risk to develop MS (International Multiple Sclerosis Genetics Consortium, 2019). In their opinion article, Ferrè et al. provided an update of genetic factors associated with MS onset, progression and treatment response. At present, the majority of genes and biological processes associated with a higher MS risk are implicated in immune functions (International Multiple Sclerosis Genetics Consortium, 2019). Conversely, only a few genetic loci involved in oligodendrocyte maturation have been suggested to contribute to MS occurrence (Factor et al., 2020). Accordingly, dysregulation of immune responses, promoted by the genetic background and potentially triggered by environmental factors, could represent the main mechanism in MS onset and progression.

Of the variety of molecular pathways involved in MS origin, several emphasize a central role of the immune system. Misrielal et al. review the role of autophagy, whereas Morgan et al. underline the role of the complement system in MS pathophysiology. The novelty resides in the completely renewed look that authors gave to these two processes in MS pathogenesis. Both autophagy and complement production could be equally supportive of either a primary immune attack against myelin or a primary response to a CNS cytodegenerative process, paving the way for future experiments that can potentially unveil the real cause of MS.

Interestingly, both autophagy and complement production may hold consequences for the stability of mitochondria, whose role in MS is not new (Witte et al., 2014). Recent attention has been dedicated to these organelles due to their putative involvement in the destabilization of the newly described axon-myelinic synapse (AMS), a dynamic form of communication between axon and myelin whose malfunction has been proposed to explain the origin of different neurodegenerative conditions (Micu et al., 2018), including MS (Luchicchi et al., 2021). In line with this notion, two articles by Bergaglio et al. and Poertoawmodjo et al. focused on the role of mitochondria instability as primitive trigger of MS pathology from two different angles: the role of oxidative stress-derived mitochondrial impairment and the possible interplay between mitochondria and Ca^2+^-dependent cysteine proteases.

Pathological studies and experimental models are the gold standard approaches to investigate MS pathophysiology. However, recently, several laboratory, neuroradiological and neurophysiological tools have been applied to define reliable biomarkers for understanding MS development and progression *in vivo*. Promising biomarkers have been presented in the two original research articles of our collection. Cennamo et al. applied Optical Coherence Tomography to investigate the role of peripapillary vessel density as an early biomarker in MS. This tool was relevant for identifying patients in the earliest phases of MS. Todea et al. showed how magnetic resonance imaging (MRI) enables identification of focal demyelination in white matter and cortical lesions already from the earliest phases of MS. Lesion burden was associated with serum neurofilament light chain (sNfL) levels, a biomarker of axonal injury. Moreover, the 2-year longitudinal changes of cortical and white matter lesion burden correlated with the cognitive performances of MS patients. This underlined the relevance of MRI biomarkers and sNfL to identify from the earliest disease phases reliable markers of neurodegeneration, disease severity and progression.

In conclusion, this issue of *Frontiers in Cellular Neuroscience*, -*Immunology*, and -*Neurology* provides an integrated overview of the “*hot topics*” in the field of MS cause. Emerging from the article collection is a complex picture where a dichotomy between outside-in/inside-out theories is replaced by a more integrated vision where both theories might equally apply according to the specific condition. The studies published in this issue emphasize the need for a re-evaluation of cellular processes, previously regarded as pure indicators of immune attack (e.g., complement), in combination with the individual (genetic) variability of MS patients, and the development of highly predictive experimental models/accurate biomarkers to unravel the unknown cause of the MS in the coming years.

## Author Contributions

AL, PP, and B'tH wrote the manuscript. All authors contributed to the article and approved the submitted version.

## Conflict of Interest

The authors declare that the research was conducted in the absence of any commercial or financial relationships that could be construed as a potential conflict of interest.
